# Circular RNA circRHOBTB3 acts as a sponge for miR-654-3p inhibiting gastric cancer growth

**DOI:** 10.1186/s13046-019-1487-2

**Published:** 2020-01-13

**Authors:** Guangxu Deng, Tingyu Mou, Jiayong He, Da Chen, Daojun Lv, Hao Liu, Jiang Yu, Shuang Wang, Guoxin Li

**Affiliations:** 1 Department of General Surgery, Nanfang Hospital, Southern Medical University, Guangdong Provincial Engineering Technology Research Center of Minimally Invasive Surgery, Guangzhou, 510515 China; 2 Department of Urology, Nanfang Hospital, Southern Medical University, Guangzhou, 510515 China; 3 Department of Pathology, Nanfang Hospital, Southern Medical University, Guangzhou, 510515 China; 4 Department of Pathology, School of Basic Medical Science, Southern Medical University, Guangzhou, 510515 China

**Keywords:** circRHOBTB3, miR-654-3p, p21, Gastric cancer, Growth

## Abstract

**Background:**

Circular RNAs (circRNAs) have recently emerged as a new family of noncoding RNAs that are involved in the causation and progression of various cancers. However, the roles of circRNAs in the tumorigenesis of gastric cancer (GC) are still largely unknown.

**Methods:**

The expression profiles of circRNAs in GC were identified in open GEO database and were evaluated at the mRNA level in clinical GC samples compared with paired non-tumorous tissues. Kaplan-Meier survival curve was used to analyze the correlation of circRNA and patients’ prognosis. Subsequently, the circular structures of candidate circRNAs were validated by Sanger sequencing, divergent primer PCR, and RNase R treatments. Gain- and loss-of-function analyses were performed to evaluate the functional significance of it in GC initiation and progression. Dual-luciferase reporter and RNA pull-down assays were used to identify the microRNA (miRNA) sponge mechanism of circRNAs.

**Results:**

The expression of circRHOBTB3 was lower in GC tissues and cell lines. Downregulation of circRHOBTB3 was significantly correlated with poor differentiation and unfavorable prognosis in patients with GC. Overexpression of circRHOBTB3 in GC cells led to decreased proliferation and induced G_1_/S arrest in vitro, accompanied with inhibited xenograft tumor growth in vivo, while the opposite effects were achieved in circRHOBTB3-silenced cells. Furthermore, we demonstrated that circRHOBTB3 acts as a sponge for miR-654-3p and verified that p21 is a novel target of miR-654-3p.

**Conclusion:**

Taken together, this study revealed that circRHOBTB3 might function as competing endogenous RNA (ceRNA) for miR-654-3p, which could contribute to growth inhibition of GC through activating p21 signaling pathway. Our data suggested that circRHOBTB3 would serve as a novel promising diagnosis marker and therapeutic target for GC.

## Background

Gastric cancer (GC) is one of the most common cancers and the third leading cause of cancer-related death worldwide [1] . Although in recent years, development of surgical treatment and adjuvant therapies significantly improves the prognosis of GC patients, the mortality rate remains high, mainly due to the heterogeneity and complicated regulatory relation at molecular level [2–5]. Hence, better understanding of the underlying molecular mechanism in GC tumorigenesis and progression is urgently needed.

CircularRNAs (circRNAs) are a novel class of endogenous non-coding RNAs mainly formed by the mechanism of “direct back splicing” or “Exon skipping” through producing a covalently closed loop, which usually originates from exon of genes [6, 7]. Although being discovered for about 40 years, the pathological and physiological process of circRNAs remains largely unknown [8–10]. With the advent of high-throughput sequencing technique and novel bioinformatic analysis thousands of circRNAs have been successfully identified in multiple cell lines and across various species. These circRNAs contain many salient features including high stability, evolutionary conservation and tissue-specific or cell type-specific expression [7, 11, 12]. Moreover, several studies have found that many circRNAs were aberrant expressed and could involve in gene regulation rather than by-products of splicing or splicing errors. Also, evidences showed that circRNAs may act as sponges for microRNAs (miRNAs) or bind to proteins, and abnormal circRNAs expression could lead to alteration of gene products that may contribute to tumor biology including cell proliferation, apoptosis, angiogenesis and metastasis [13–16]. In fact, evidence from recent literatures and our previous studies has implied that circRNAs play a pivotal role in the tumorigenesis of GC [17–19]. These findings suggested that circRNAs might be a novel biomarker for the diagnosis and treatment of cancer. However, these studies only exhibited the preliminary results on the circRNA-miRNA regulatory network in GC. The overall pathophysiological role of circRNAs in GC needs to be further investigated.

In the present study, we identified one circRNA originating from exon 6 and exon 7 of RHOBTB3 gene and termed it circRHOBTB3. Meanwhile, the expression of circRHOBTB3 was determined in paired GC tissues and the relationship between circRHOBTB3 with patients’ clinicopathologic characteristic was also analyzed. Besides, the functions of circRHOBTB3 in the growth of GC were explored both in vitro and in vivo. Finally, we confirmed that circRHOBTB3 could sponge miR-654-3p and promoted the expression of p21, a major effector molecule of the cell cycle inhibiting protein.

## Materials and methods

### Patients and tissue specimens

Seventy-five pairs of GC tissue and paired non-cancerous tissue were obtained from patients with GC who had endoscopically proven primary GC and received radical surgical treatment at Nanfang Hospital (Guangzhou, China) between 2017 and 2019. All patients enrolled in this study did not receive prior surgery, radiotherapy or chemotherapy. All specimens were immediately snap-frozen in liquid nitrogen before storage at − 80 °C. Fifty nine samples with clinico-pathological parameters was used to analyze the significance and prognostic value of circRHOBTB3. Written informed consent was obtained from all patients. The procedure of human tissue samples collection was conducted in accordance with the international Ethical guidelines for biomedical research involving in human subjects. This study was approved by Southern Medical University Ethics Committee.

### Cell culture

Human GC cell lines (AGS, HGC27, MKN45) were obtained from the Committee of Type Culture Collection of Chinese Academy of Sciences (Shanghai, China). In addition, the gastric mucosal cell GES-1 was provided by the Department of Pathology, Nanfang Hospital, Southern Medical University. The cells were cultured in RPMI Medium 1640 (Gibco, Australiaorigin) supplemented with 10% FBS (BI, China) at 37 °C in a humidified atmosphere of 5% CO_2_.

### RNA preparation, qRT-PCR and genomic DNA purity

Total RNA was extracted from tissues and cells by using TRIzol reagent (Takara, Otsu, Japan). Total RNA was reverse transcribed to cDNA and qRT-PCR were conducted by using a SYBR Green PCR Kit (Takara, Otsu, Japan) as described [20, 21]. For miRNA detection, reverse transcription was performed and expression of miRNA was measured by All-in-One™ miRNA qRT-PCR Detection Kit (Genecopoaie, Lot#QP015) according to the use manual. Genomic DNA (gDNA) was isolated from tissues or cultured cells according to easy pure genomic DNA kit (Transgen Biotech, Lot#L61221). All primers were listed in Additional file 2: Table S1.

### Nucleic acid electrophoresis

The cDNA and gDNA PCR products were detected using 4% agarose gel electrophoresis with TAE running buffer at 100 V for 30 min. DNA marker used was DL2000 (Takara, Japan). The bands were examined by UV irradiation.

### Treatment with RNase R and Actinomycin D

Total RNA (2 μg) was incubated for 15 min at 37 °C with or without 3 U/mg of RNase R (Epicentre Technologies, Madison, WI, USA). Then, the resulting RNA was directly reverse transcribed using the prime script RT master mix (Takara, Japan) for real-time PCR analyses. To assess the stability of circRHOBTB3 and its linear isoform, The culture medium was added to Actinomycin D or DMSO (sigma Aldrich, St. Louis, MO, USA) as described [18].

### Cell proliferation and cell cycle

Cell proliferation was examined using cell counting kit-8 assay (Dojindo Laboratories, Kumamoto, Japan) and the cell-light™ Edu staining kit (RiboBio, Guangzhou, China) according to manufacturer’s instructions. Colony formation assay were performed to monitor cell cloning capability as followed: 1 × 10^3^ cells was seeded into 6-wells plates and cultured at 37 °C in atmosphere of 5% CO_2_ for 2 weeks. Then the colonies were washed twice with PBS, fixed with 4% paraformaldehyde for 10 min and dyed with Wright-Giemsa Stain. The number of colonies was photographed and counted using a microscope. Cell cycle was analyzed by flow cytometry. In brief, cells were collected, fixed with cold ethanol for 2 h at 37 °C, washed by PBS, stained with propidium iodide (PI) (Keygentec, Nanjing, China) containing RNase A and then the cell cycle was detected by a flow cytometer (FACS Calibur, Becton Dickinson).

### Fluorescence in situ hybridization (FISH)

Cy3-labled probes including circRHOBTB3 and 18 s and FAM-labled locked nucleic acid miRNA probes were designed and synthesized by RiboBio (Guangzhou, China) and GenePharm (Suzhou, China), respectively, and the probe sequences were obtained on request. The signals of the probes were detected by Fluorescence In Situ Hybridization kit (RiboBio, Guangzhou, China) according to the manufacturer instruction. The images were acquired on Nikon AISi Laser Scanning Confocal Microscope (Nikon instruments Inc., japan).

### Oligonucleotide transfection

SiRNA or miRNA mimic was designed and synthesized by Suzhou GenePharma (Suzhou, china) and RioBio (Guangzhou, china), respectively. The sequences used were shown in Additional file 2: Table S1. The cells were transfected using lipofectamine2000 (Thermo Fisher, Shanghai, China).

### Over-expressing plasmids construction and stable cells transfection

To establish circRHOBTB3 over-expression plasmids, human circRHOBTB3 full-length cDNA was synthesized by vigenebio (Shandong, China) and inserted into PKO-ciR vector including a front circular frame and a back circular frame. Transfection was carried out using lipofectamine2000 (Thermo Fisher, Shanghai, China) according to manufacturer’s instructions. Stable over-expression circRHOBTB3 cells were constructed using over-expressing circRHOBTB3 lentivirus carrier (Vigenebio, Shandong, China). We designed two small interfering RNA (siRNAs) targeting the junction sites of circRHOBTB3 to silence circRHOBTB3 expression in AGS and HGC27 cell lines. These siRNA could significantly knock down circRHOBTB3 levels without affecting its linear isoform, and we selected si-circRHOBTB3–1 to insert into lentivirus carrier to establish stable silencing circRHOBTB3 cell lines due to its higher inhibitory efficacy of circRHOBTB3.

### Western blot

Western blot analysis was performed as illustrated [22–24]. In brief, cells were harvested, lysed with radio immunoprecipitation assay buffer (RIPA, Beyotime, China), and quantified by bicinchoninic acid (BCA) analysis (Beyotime, China). Then, protein extractions were separated by 10% SDS-PAGE, transferred onto polyvinylidene fluoride (PVDF) membranes (millipore Corporation, Billercica, MA, USA), washed by TBST, and incubated with a high affinity anti-p21 antibody (1:1000), and anti-Tubulin antibody (1:1000) (Cell Signaling Technology, USA) overnight at 4 °C. Subsequently, the membranes were incubated with a secondary antibody (Cell Signaling Technology, USA) (1:5000). After washes, signals were visualized by the enhanced chemiluminescence (ECL) detection system (Pierce Biotechnology, Rockford, IL, USA) conducting in accordance with the manufacturer’s instructions.

### Biotinylated RNA pull-down assay

Pull down assay was carried out as described [25]. In brief, for circRHOBTB3 pull down miRNA, about 1 × 10^7^ cells were harvested, lysed and sonicated. The circRHOBTB3 probe was incubated with streptavidin magnetic beads (Beaver, Suzhou, china) for 2 h to generate probe-coated beads, then incubated with cell lysates, followed by eluted with Trizol (Takara, japan), and qRT-PCR. for miR-654-3p pulled down, about 1 × 10^7^ cells were collected, lysed, sonicated, and incubated with streptavidin magnetic beads (beaver, Suzhou, china) after transfected with biotinylated miR-654-3p mimics or mutant using lipofectamine2000 (Thermo Fisher, Shanghai, China), followed by washed, eluted and qRT-PCR.

### Luciferase reporter assays

For circRHOBTB3 and miRNA luciferase assay, the circRHOBTB3 sequences containing wild-type or mutated miRNA binding sites were respectively synthesized and inserted into pEZX-MT06 luciferase vector (Genecopoaie, Guangzhou, China), and then co-transfected with miRNA mimics into GC cell lines using lipofectamine2000 (Thermo Fisher, Shanghai, China). After 48 h transfection, cells were harvested, lysed, and subjected to luciferase activity detection by the Luc-pair™ Duo-Luciferase HS assay kit (Genecopoaie, Guangzhou, China). Relative luciferase activity was normalized to the Renilla luciferase internal control.

### Animal experiments

All animal experiments were approved by the Animal Care Committee and Use Committee of Southern Medical University. The experiments were in accordance with the guidelines for the ethical treatment of animals. To investigate the circRHOBTB3 growth effect on GC cells in vivo, 4-week-old male BALB/C nude mice were randomly divided into two groups (*n* = 5 for each group). The stable over-expressed circRHOBTB3 MKN45 cells or control group were subcutaneously injected into flank region of legs (5 × 10^6^ cells per mouse), respectively. Tumor size were measured from perpendicular axes and calculated as followed formula: volume = (length×width^2^)/2. One month later, mice were sacrificed by cervical vertebra dislocation. The primary tumors were removed and harvested, and weighed. Then, the tumors were fixed, paraffin-embedded and sectioned. Subsequently, the sections were visualized under a microscope following hematoxylin and eosin (H&E) staining.

### Immunohistochemistry

Immunohistochemical staining was performed as described [26]. Slides from nude mice were incubated overnight with primary antibodies against ki67 (#ab15580, Abcam), p21 (#ab15580, Abcam) at 4 °C. The complex was observed by DAB complex, and the nuclei were counterstained with haematoxylin. The immunoreactivity in each section was assessed by at least two experienced pathologists and scored by semi-quantitative H-score approach [27].

### Statistical analysis

Statistical analyses were performed using SPSS 20.0 software (IBM, Armonk, NY, USA) and Graphpad prism version 5.0 (Graphpad software, La Jolla, CA, USA). Data was expressed as mean ± SD. The statistical differences between groups were tested by two-tailed student’s *t-test*. The correlation between the circRHOBTB3 expression and clinicopathologic data in GC tissues and paired normal tissues were examined by χ^2^ test. The relationship between the circRHOBTB3 and linear RHOBTB3 or miR-654-3p was examined by Pearson’s correlation coefficients. Survival prognosis was analyzed by Kaplan-Meier curves and log-rank test for significance. *p* < 0.05 was considered statistically significant.

## Results

### CircRHOBTB3 was significantly downregulated in GC tissues, cell lines and correlated with prognosis of patients with GC

**Fig. 1:**
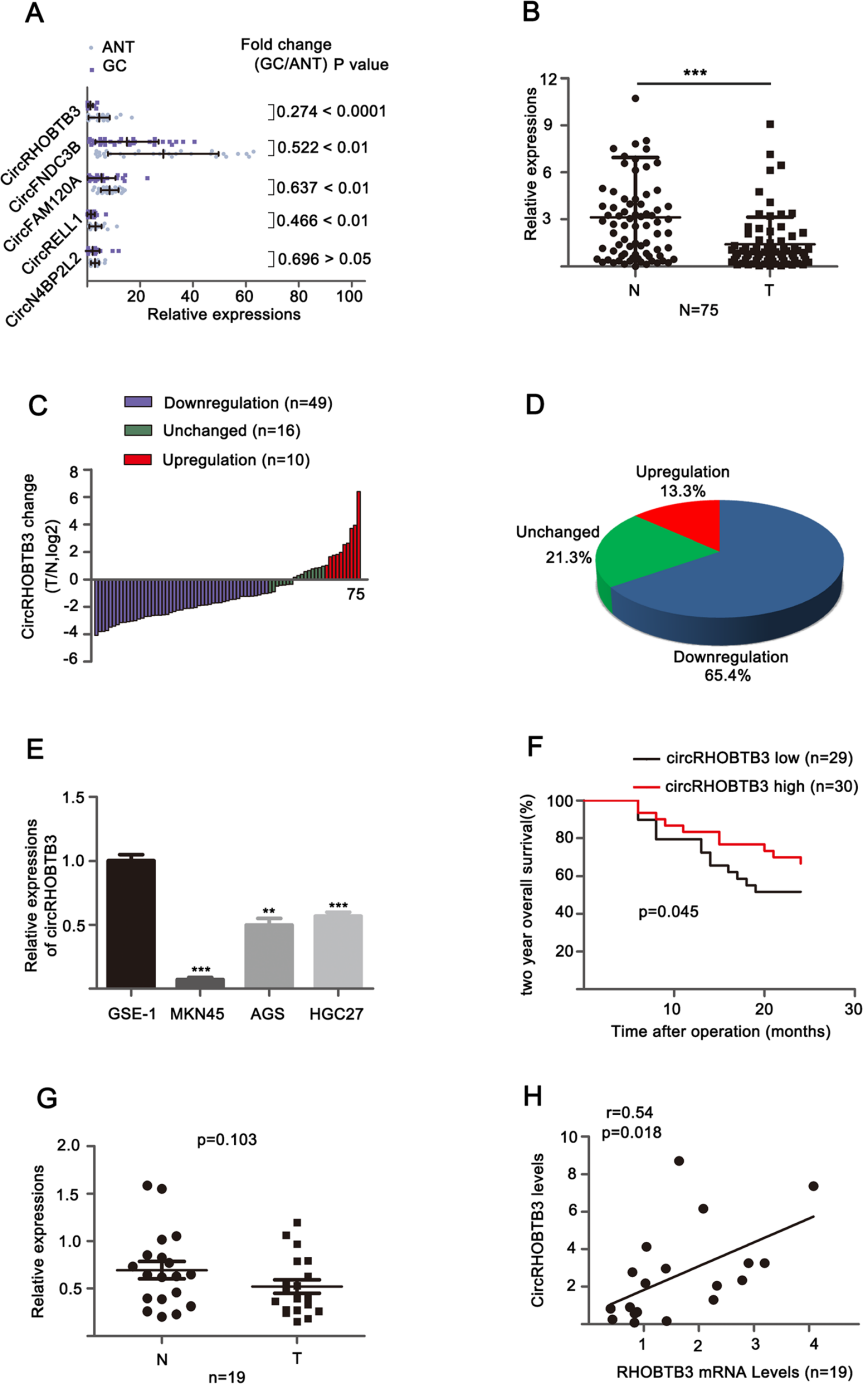
CircRHOBTB3 was frequently repressed in GC and correlated with prognosis of patients with GC. **a** qRT-PCR for determining relative levels of five circRNAs in GC and normal tissues (*n* = 30). **b** qRT-PCR for expression of circRHOBTB3 in GC tissues and adjacent normal tissues(*n* = 75). **c** A fold change analysis of circRHOBTB3 in 75 paired GC tissues and matched normal tissues. GAPDH was used as internal control. **d** Pie chart of the proportion of GC sample in which circRHOBTB3 expression was downregulated (blue), unchanged (green) and upregulated (red). **e** qRT-PCR detection of circRHOBTB3 expression in GC cells (AGS, MKN45, HGC27) and GES-1 cells (the normal gastric mucosal cells). GAPDH was served as internal control. **f** Kaplan-Meier analysis of relationship between expression of circRHOBTB3 and GC patients’ overall survival. **g** The expressed profile of RHOBTB3 mRNA was quantified by qRT-PCR in 19 GC and ANT. **h** The relationship between circRHOBTB3 and its mother RHOBTB3 gene evaluated by Pearson’s correlation coefficients in GC and normal tissues(*n* = 19). The data was expressed as the mean ± SD and reproduced in three independent experiments. GC: gastric cancer. ANT: adjacent normal tissue *p < 0.05, **p < 0.01, ***p < 0.001

To identify a candidate circRNA that may implicate in the tumorigenesis of GC, we firstly analyzed the high-throughput sequencing results of GC (accession code: GSE77661) from the open GEO database [18]. Then we selected five candidate circRNAs from the RNA-seq data by the criteria: fold change > 2 (Circbank numbers: circN4BPL2L, circRELL1, circFAM120A, circFNDC3B, circRHOBTB3). Moreover, the relative expression of these five circRNAs were detected by qRT-PCR in GC and paired non-tumorous tissues (cohort 1, *n* = 30). Results showed that the relative expressions of circN4BPL2L displayed no significant changes, but the other four circRNAs were low-expressed. Interestingly, we found that circRHOBTB3 was the most significantly downregulated circRNA among them (*P* < 0.0001 and fold-change (GC/ANT) < 0.274, Fig. 1a), which suggested that circRHOBTB3 might be a circular molecular correlated with progression of GC.

To further confirm the relationship between the expression level of circRHOBTB3 and the clinical progress of GC patients. qRT-PCR was used to detected the expressions of circRHOBTB3 in newly internalized GC tissues and adjacent tissues (cohort 2, *n* = 75). Results displayed that circRHOBTB3 was frequently repressed in GC tissues compared with non-tumorous tissues (Fig. 1b). When primary cancers were paralleled to corresponding normal tissues, the downregulation of circRHOBTB3 (less than a 0.5-fold change) was observed in 65.4% (49/75) of patients with GC (Fig. 1c, d). Consistently, we also found that the expression of circRHOBTB3 in GC cell lines was obviously downregulated compared with GES-1 cells (the normal gastric mucosal cells) (Fig. 1e). Subsequently, these patients were stratified into high and low groups based on the median value of circRHOBTB3 expression. The relationship between circRHOBTB3 expression in GC tissues and clinicopathological features. As shown in Table 1, low expression of circRHOBTB3 was dramatically correlated with tumor stage, whereas no significant correlation with other clinicopathologic parameters. In addition, survival analyses of these patients revealed that patients with low expression of circRHOBTB3 had poor prognosis in GC than patients in the high circRHOBTB3 expression group (Fig. 1f). These collective data suggested that circRHOBTB3 might play pivot role in the tumorigenesis of GC.

**Table 1:** Correlation between circRHOBTB3 expression and clinicopathologic characteristics in GC patients

Characteristics	CircRHOBTB3^a^ expression			
	n	Low	High	*P* value
Age				
< 60	31	11 (39.3%)	20 (64.5%)	0.053
≥ 60	28	17 (60.7%)	11 (35.5%)	
Gender				
Male	41	21 (75.0%)	20 (64.5%)	0.382
Female	18	7 (25%)	11 (35.5%)	
Tumour size (cm in diameter)				
≤ 5	47	22 (78.6%)	25 (80.6%)	0.843
>5	12	6 (21.4%)	6 (19.4%)	
Location				
Upper	13	5 (17.9%)	8 (25.8%)	0.725
Middle	10	4 (14.3%)	6 (19.4%)	
Lower	33	17 (60.7%)	16 (51.6%)	
Whole	3	2 (7.1%)	1 (3.2%)	
Histological stage				
Well/moderate	16	10 (35.7%)	6 (19.4%)	0.158
Poor/undifferentiated	43	18 (64.3%)	25 (80.6%)	
Vascular invasion				
No	36	19 (67.9%)	17 (54.8%)	0.306
Yes	23	9 (32.1%)	14 (45.2%)	
AJCC stage				
I/II	18	13 (46.4%)	5 (16.1%)	0.012*
III/IV	41	15 (53.6%)	26 (83.9%)	
T stage				
T1–3	10	7 (25.0%)	3 (9.7%)	0.223
T4	49	21 (75.0%)	28 (90.3%)	
N stage				
N0	39	19 (67.9%)	20 (64.5%)	0.787
N1–3	20	9 (32.1%)	11 (35.5%)	
M stage				
M0	57	26 (92.9%)	31 (100%)	0.221
M1	2	2 (7.1%)	0 (0.0%)	

*p < 0.05

**p < 0.01

^a^Using circRHOBTB3 median value as cutoff, the log(2_,_ X) value < cutoff was low expression and the log(2_,_ X) value ≥ cutoff was high expression. X, GC/ANT

It has been reported that some circRNAs may modulate the corresponding linear RNA transcripts expression and then execute function [28, 29]. Therefore, the regulatory relationship between circRHOBTB3 and its linear RNA transcript (RHOBTB3) was explored. Firstly, the expression level of RHOBTB3 was examined in the 19 paired GC and adjacent non-tumorous tissues (Fig. 1g). However, no significant changes of RHOBTB3 mRNA was observed. Pearson’s correlation analysis revealed a significant positive correlation between circRHOBTB3 and its linear RHOBTB3 in GC tissues (r = 0.54, *P* = 0.018, Fig. 1h). Nevertheless, RHOBTB3 did not change the mRNA expression levels when the expression of circRHOBTB3 was artificially changed in GC cells (Additional file 1: Figure S2A-C). These results indicated that RHOBTB3 is not the target gene of circ RHOBTB3.

### Characteristics of circRHOBTB3

**Fig. 2:**
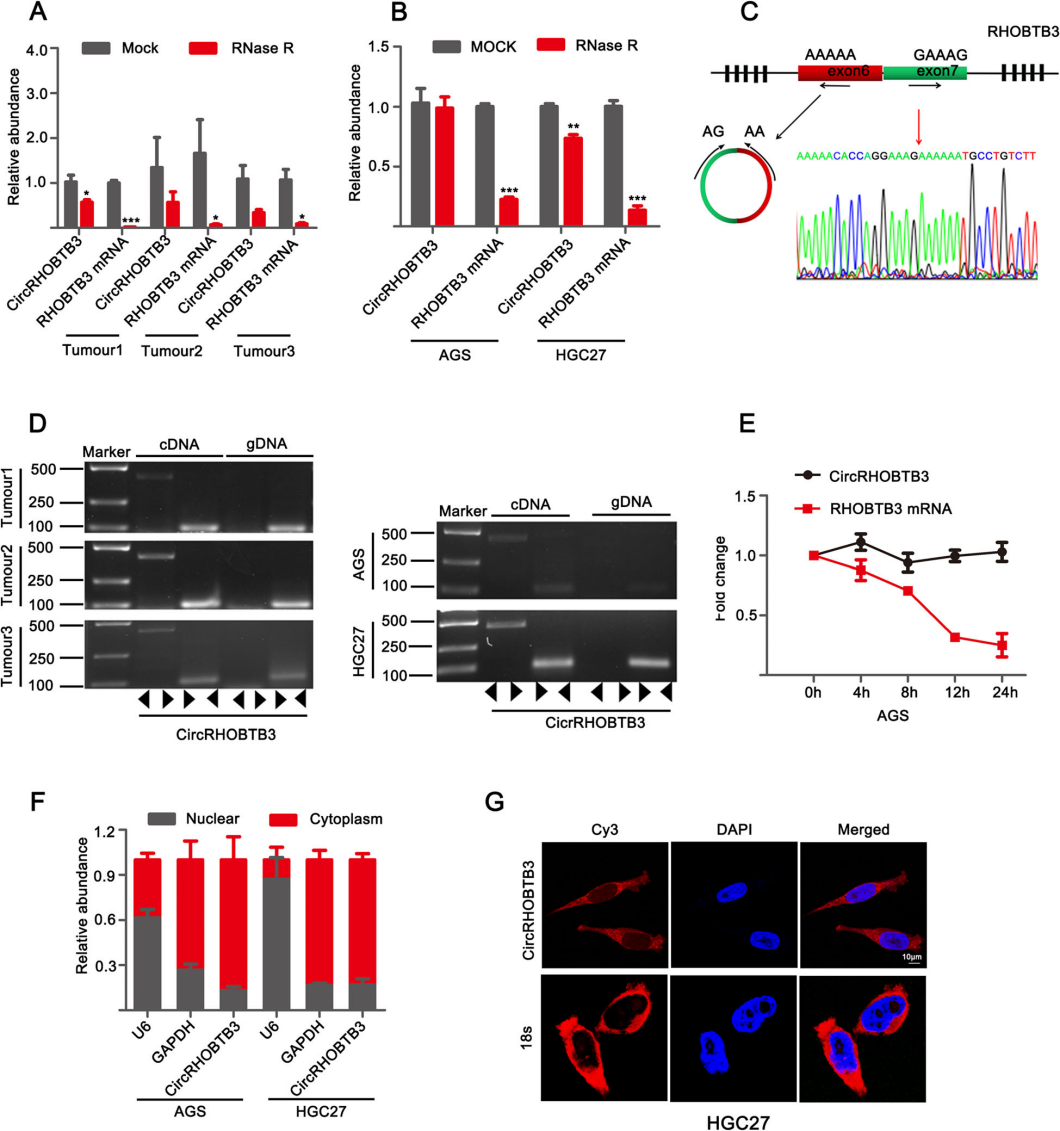
Characters of circRHOBTB3. **a** The relative circRHOBTB3 or linear RHOBTB3 mRNA abundance detected by qRT-PCR after treated with or without RNase R in three GC tissues. **b** qRT-PCR for the relative abundance of circRHOBTB3 and RHOBTB3 mRNA in AGS and HGC27 cell lines after treated with RNase R. The amount of circRHOBTB3 and RHOBTB3 mRNA were standardized to the value detected in the mock treatment. **c** The constitutions of circRHOBTB3 formed by exon6 and exon7 of RHOBTB3 gene illustrated by the schematic diagram. The sequence of back-junction of circRHOBTB3 was validated by sanger sequencing. Red arrow showed the “head-tail” splicing sites of circRHOBTB3. **d** CircRHOBTB3 verified in three GC tissues and AGS and HGC27 cell lines by RT-PCR. CircRHOBTB3 amplified by divergent in cDNA but not in genomic DNA (gDNA). **e** qRT-PCR for abundance of circRHOBTB3 and RHOBTB3 mRNA in AGS cell line treated with Actinomycin D at indicated time point. **f** qRT-PCR value indicating the abundance of circRHOBTB3, U6 and GAPDH in either the cytoplasm or nuclear of AGS and HGC27 cell lines. GAPDH and circRHOBTB3 were normalized to the value measured in cytoplasm. U6 was normalized to the value measured in nuclear. **g** RNA FISH was conducted to detect circRHOBTB3’s subcellular in HGC27 cell lines. Nuclei was stained with DAPI. 18 s probe was served as positve control. Scale bar, 10 μm

CircRHOBTB3 was generated from exon 6 and exon7 of RHOBTB3 gene (CircBase ID: hsa_circ_00074444, splicing length: 479 nucleic acid base). To further confirm circular characteristics of circRHOBTB3, the transcripts of both circRHOBTB3 and RHOBTB3 mRNA was tested by qRT-PCR in three tumor tissues, AGS and HGC27 cell lines after treatment with or without RNase R. Results showed that the fragment of linear form of RHOBTB3 gene was digested by RNase R while cirRHOBTB3 was retained after RNase R treatment (Fig. 2a, b), which verified that circRHOBTB3 was resistant to RNase R due to its loop structure. Secondly, to rule out the possibility of head-to-tail sequencing produced by trans-splicing or genomic rearrangement, Divergent primers and convergent primers were designed to amplify circRHOBTB3 and RHOBTB3 mRNA, respectively. cDNA and gDNA (genomic DNA) from three GC tissues and AGS, HGC27 cell lines were used as templates. We found that circRHOBTB3 was only amplified by divergent primers in cDNA, but no amplification product was visualized in gDNA. Meanwhile, the head-to-tail junction sequences were validated by Sanger sequencing (Fig. 2c, d). Then, inhibiting transcription experiment was utilized to reveal the stability of circRHOBTB3, and illustrated that it was more stable than its linear mRNA (Fig. 2e). Additionally, the subcellular localization of circRHOBTB3 was determined in nucleoplasmic separation and FISH experiments. Results indicated that circRHOBTB3 was preferentially localized in cytoplasm (Fig. 2f, g and Additional file 1: Figure S1). Taken together, the above results indicated that circRHOBTB3 is an abundant, circular and stable transcript that mainly localized in cytoplasm of GC cells.

### CircRHOBTB3 inhibited GC cell growth and cell cycle progression in vitro

**Fig. 3:**
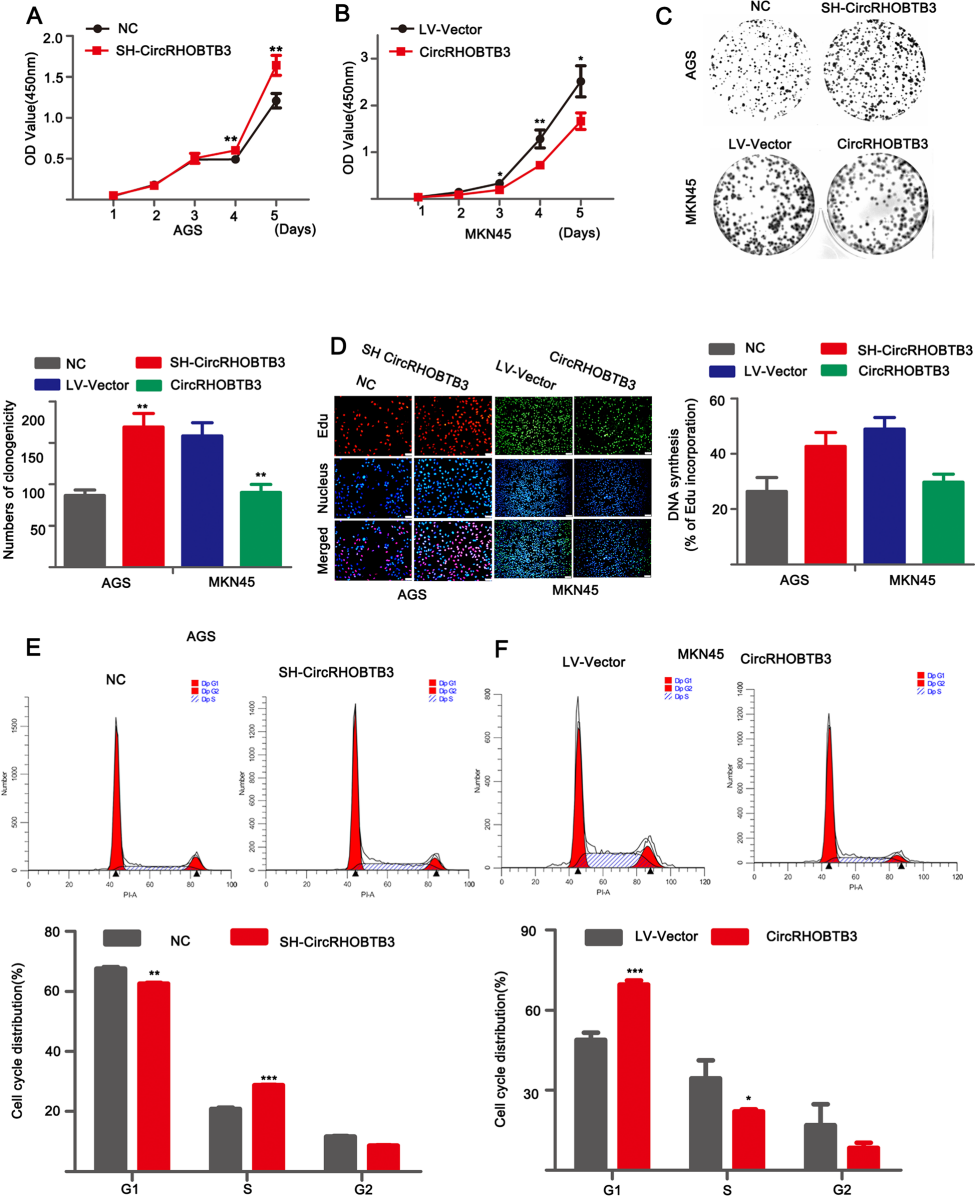
CircRHOBTB3 inhibited proliferation of GC cells in vitro. **a**, **b** cck8 assay of AGS transfected with control lentivirus (NC) or LV-SH-circRHOBTB3 (SH-circRHOBTB3) and MKN45 transfected with lentivirus vector or over-expressed circRHOBTB3 letivirus. **c**, **d** Colony formation assay and Edu assay were conducted to evaluated cell proliferative ability in stable silenced circRHOBTB3 AGS cells or over-expressed circRHOBTB3 MKN45 cells. **e**, **f** Representative images of cell cycle in AGS cells after silencing circRHOBTB3 or MKN45 cells after elevating circRHOBTB3 were analyzed by flow cytometry. The data was expressed as the mean ± SD and reproduced in three independent experiments. *p < 0.05, **p < 0.01, ***p < 0.001

To better understand the role of circRHOBTB3 in GC cells. We selected si-circRHOBTB3–1 to insert into lentivirus carrier to establish stable silencing circRHOBTB3 (SH-circRHOBTB3) in AGS and HGC27 cell lines due to its higher inhibitory efficacy of circRHOBTB3. Data demonstrated that stable SH-circRHOBTB3 AGS and HGC27 cell lines were established successfully (Additional file 1: Figure S2A, B). Moreover, circRHOBTB3 were stably over-expressed by circRHOBTB3-overexpressed lentivirus vector in MKN45, AGS and HGC27 cells lines (Additional file 1: Figure S2C). Subsequently, functional assays were executed to reveal the effects of circRHOBTB3 on GC cell proliferation. The cck8 assay showed that knocked-down of circRHOBTB3 enhanced the growth ability of AGS and HGC27 cell lines significantly (Fig. 3a and Additional file 1: Figure S2D). Whereas, over-expression of circRHOBTB3 suppressed the proliferation of MKN45 cells (Fig. 3b). In addition, the colony formation assay indicated that SH-circRHOBTB3 AGS and HGC27 cell lines produced more clonogenicities compared with control group but elevation of circRHOBTB3 displayed opposite effect in MKN45 cells (Fig. 3c and Additional file 1: Figure S2E). Consistently, by using Edu assay, compared with control group, more proliferative cells were observed in AGS and HGC27 cell lines with down-regulation of circRHOBTB3 while less in MKN45 cells with up-regulation of circRHOBTB3 (Fig. 3d and Additional file 1: Figure S2F). Moreover, Flow cytometry analysis was performed to determine whether circRHOBTB3 affected the cell cycle profile. As presented in Fig. 3e and Additional file 1: Figure S2G, less cells stopped in G1 phase after silencing of circRHOBTB3 in AGS and HGC27 cell lines, which suggested that silencing of circRHOBTB3 induced G_1_/S cell cycle progression. However, over-expression of circRHOBTB3 arrested cell cycle in G1 phase in MKN45 cells (Fig. 3f). These data collectively demonstrated that circRHOBTB3 inhibited the growth phenotype of GC cells and cell cycle progression.

### CircRHOBTB3 acted as a molecule sponge for miR-654-3p

**Fig. 4:**
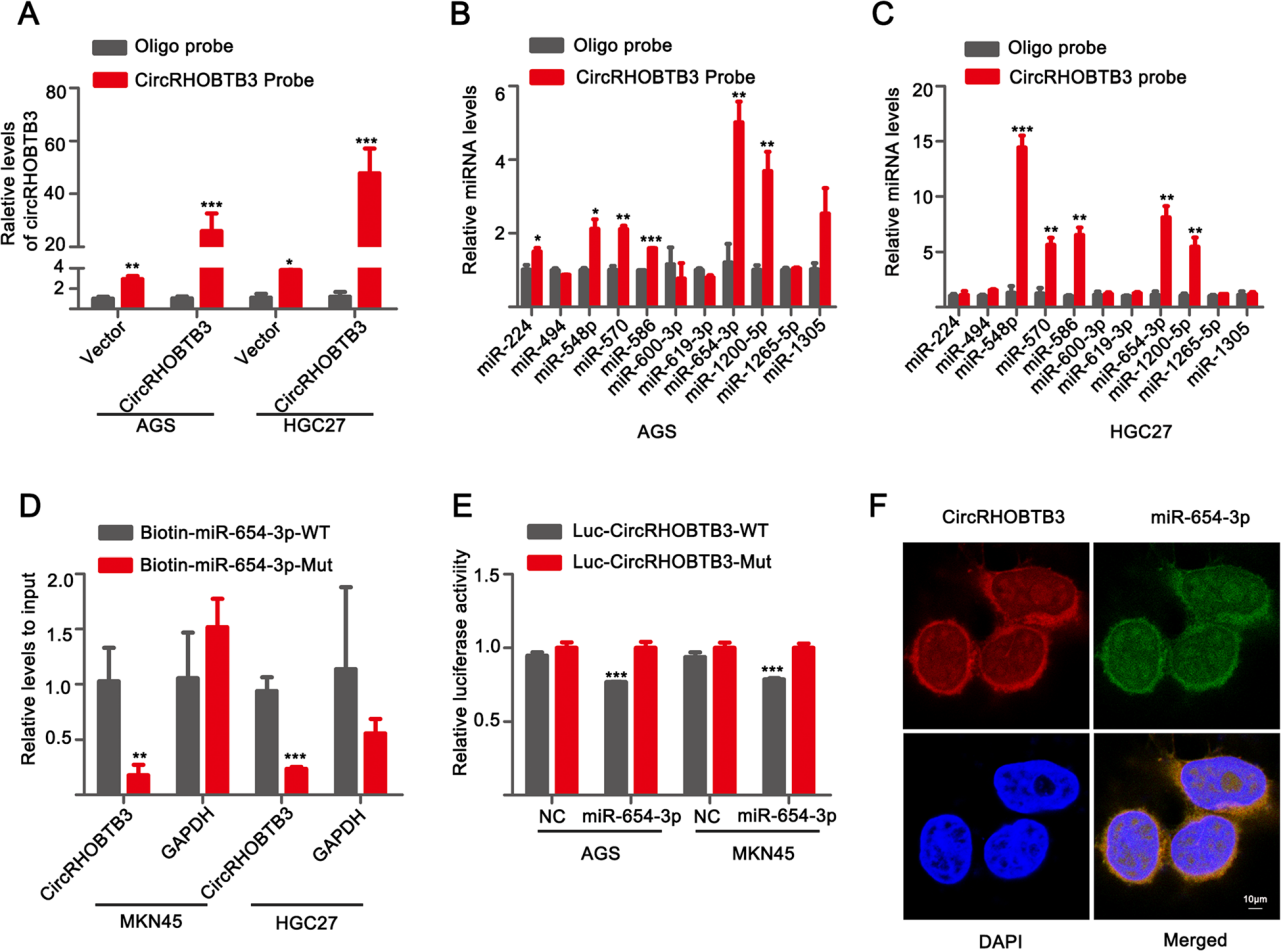
CircRHOBTB3 served as a sponge for miR-654-3p in GC cell lines. **a** Lysates from AGS and HGC27 cell lines transfected with plasmid-vector or plasmid-circRHOBTB3 were subjected to biotinylated-circRHOBTB3 pull-down assay and the expression levels of circRHOBTB3 were measured by qRT-PCR. **b**, **c** The expression levels of top eleven candidate miRNAs predicted by Circinteractome database were quantified by qRT-PCR after biotinylated-circRHOBTB3 pull-down assay in AGS and HGC27 GC cells. **d** Biotin-coupled miR-654-3p wild type (biotin-miR-654-3p-WT) or its mutant (biotin-miR-654-3p-Mut) captured relative expressions of circRHOBTB3 in the complex. GAPDH was used as negative control. Relative levels of circRHOBTB3 or GAPDH was normalized to input. **e** Luciferase activity was tested in AGS and MKN45 cells co-transfected with luciferase reporter containing circRHOBTB3 sequences with wild type and mutant binding site of miR-654-3p and the mimic of miR-654-3p or control. **f**, **g** Fluorescence in situ hybridization was performed to examine the co-location between circRHOBTB3 and miR-654-3p in AGS cell lines. Scale bar = 10 μm. The data was expressed as mean ± SD of at least three independent experiments. *p < 0.05, **p < 0.01, ***p < 0.001

Previous studies suggested that circRNAs mainly function as miRNA sponge to sequester miRNA and then regulate subsequent gene expression [30, 31]. To investigate whether circRHOBTB3 served as “miRNA sponge” in GC cells, we selected the eleven top potential binding miRNA (miR-244, miR-494, miR-548p, miR-570, miR-586, miR-600-3p, miR-619-3p, miR-654-3p, miR-1200-5p, miR-1265-5p, miR-1305) with a context score percentile≥90 predicted by CircInteractome database. The overexpression efficacy of circRHOBTB3 was quantified by qRT-PCR in AGS and HGC27 cell lines transfected with elevated circRHOBTB3 plasmid or empty (Additional file 1: Figure S3A). Subsequently, a 3′ terminal-biotinylated-circRHOBTB3 probe was designed to determine which miRNAs interacted with circRHOBTB3. As illustrated in Fig. 4a, the probe was confirmed to pull down circRHOBTB3 in AGS and HGC27 cell lines and overexpression of circRHOBTB3 increased pull-down efficacy. Then, qRT-PCR analyses revealed that several miRNAs including miR-548p, miR-570, miR-586, miR-654-3p and miR-1200-5p could all be pulled down in AGS and HGC27 cell lines. Among them, miR-654-3p was most abundantly pulled down by circRHOBTB3 in HGC27 and AGS cells (Fig. 4b, c). Meanwhile, miR-654-3p was reported and predicted by Target Scan website to target p21 protein, a well-known proliferative suppressor in various tumors [32–34]. Therefore, we focused on miR-654-3p for further study. To adequately consolidate the binding between circRHOBTB3 and miR-654-3p, biotin-coupled miR-654-3p and its mutant were utilized to pull down circRHOBTB3 in MKN45 and HGC27 cell lines with stable circRHOBTB3 overexpression. Results showed that wild-type miR-654-3p captured more circRHOBTB3 compared with its mutant. GAPDH served as negative control and showed no significant changes between biotin-labeled miR-654-3p and mutant group (Fig. 4d). Furthermore, we conducted luciferase reporter assay and verified that up-regulation of miR-654-3p relatively reduced the luciferase activity of vector containing full circRHOBTB3 sequences, but didn’t influence the luciferase activity of vector including mutant binding sites of miR-654-3p in AGS, HGC27, and MKN45 cell lines (Fig. 4e and Additional file 1: Figure S3B, C). Moreover, we conducted FISH assay to assess whether there was co-location between circRHOBTB3 and miR-654-3p. Results indicated that co-location of circRHOBTB3 and miR-654-3p was mainly visualized in cytoplasm (Fig. 4f and Additional file 1: Figure S3D). Based on the above, data demonstrated that circRHOBTB3 could serve as a sponge molecule for miR-654-3p.

### CircRHOBTB3 inhibits GC growth through the circRHOBTB3/ miR-654-3p/p21 pathway

**Fig. 5:**
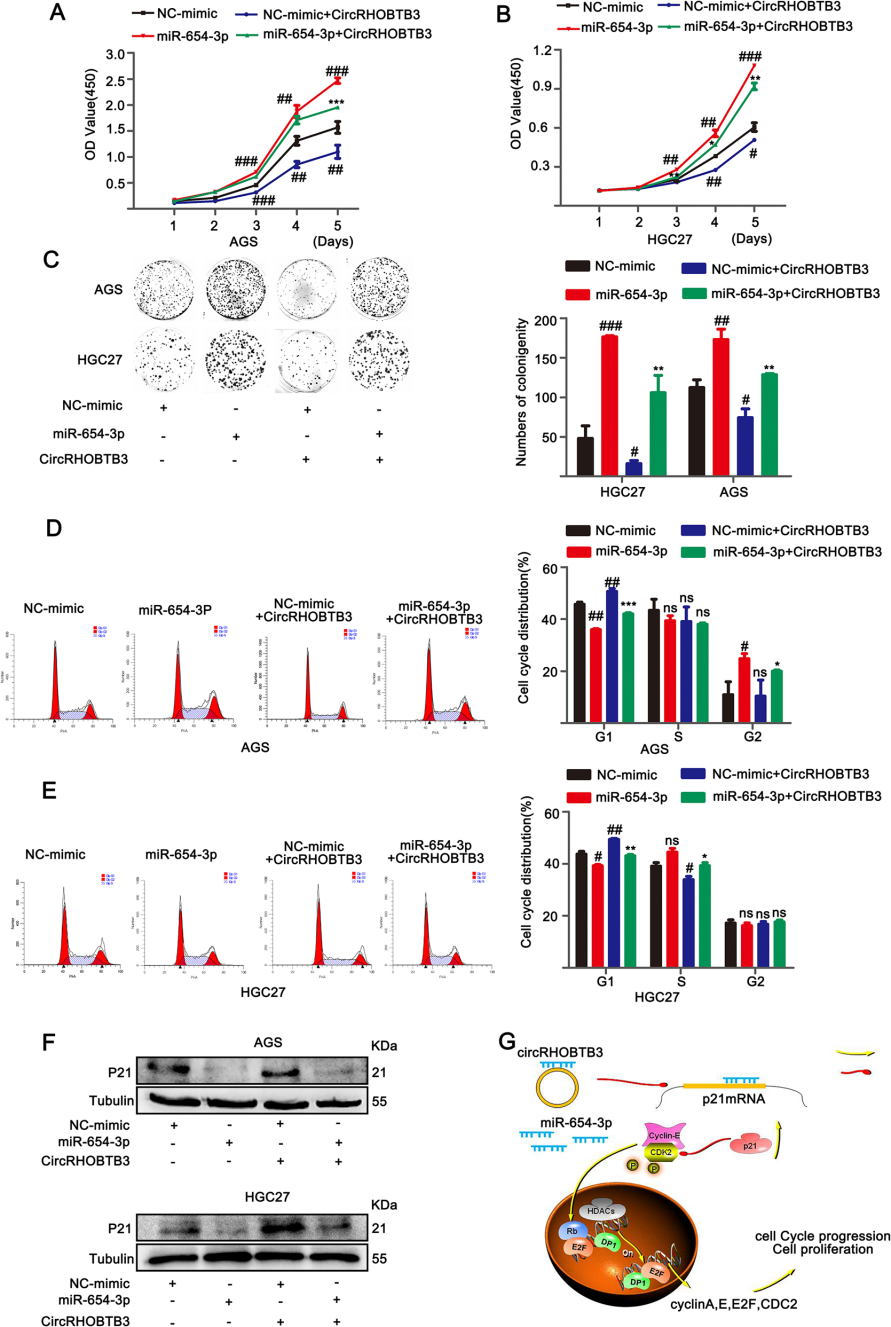
Overexpression of circRHOBTB3 partly reversed miR-654-3p-induced proliferation of GC cells. **a**, **b** cck8 assay, **c** colony formation assay were performed to evaluate the ability of proliferation in overexpressed circRHOBTB3 AGS and HGC27 cell lines transfected with miR654-3p or NC-mimiR. **d**, **e** Cell cycle distribution was analyzed by flow cytometry. ^#^ vs control group, ^*^ vs miR-654-3p group. **f** p21 protein expression was determined by western blot. **g** Mode pattern of circRHOBTB3/miR-654-3p/p21 regulatory network. The data was expressed as mean ± SD of at least three independent experiments. *p < 0.05, **p < 0.01, ***p < 0.001. ^#^*p* < 0.05, ^# #^*p* < 0.01

To address whether circRHOBTB3 plays tumor-inhibiting role via circRHOBTB3/miR-654-3p/p21 pathway in GC, we performed the following experiments: Firstly, the expression levels of miR-654-3p were detected by qRT-PCR in 30 paired GC tissues and adjacent non-cancerous tissues. Results indicated that miR-654-3p was highly expressed in GC tissues compared with control group (Additional file 1: Figure S4A). In addition, we found that the expression of miR-654-3p was negatively correlated with circRHOBTB3 in GC tissues analyzed by Pearson’s correlation (Additional file 1: Figure S4B), which cued that miR-654-3p might severe as proliferation-promoting role in GC. Consistently, the expression level of miR-654-3p was up-regulated in GC cell lines in comparison to GES-1 cells (Additional file 1: Figure S4C). Subsequently, cck8 assay was conducted, and confirmed that miR-654-3p enhanced the growth of AGS and HGC27 cell lines transfected with miR-654-3p compared with NC-mimic (Additional file 1: Figure S4D, E). Then, we examined the protein levels of p21 in AGS and HGC27 cell lines transfected with miR-654-3p or NC-mimic, and found that p21 expression was significantly repressed in miR-654-3p group compared with NC-mimic, as shown in Additional file 1: Figure S4F. Additionally, we examined the protein expression of p21 using western blot in stable over-expression or down-regulation circRHOBTB3 GC cell lines. As expected, results showed that protein expression of p21 was upregulated in AGS and HGC27 cells with stably elevated circRHOBTB3 and downregulated in SH-circRHOBTB3 AGS and HGC27 cell lines (Additional file 1: Figure S4G, H). However, qRT-PCR analyses for the mRNA levels of p21 indicated that there were no significant changes in AGS and HGC27 cell lines treated with miR-654-3p or NC-mimic or stable overexpressed circRHOBTB3 GC cell lines (Additional file 1: Figure S3I), suggesting that miR-654-3p or circRHOBTB3 didn’t affect p21 mRNA levels. To further explore whether circRHOBTB3 acted as tumor-inhibitor in GC cell by attenuating activity of miR-654-3p to up-regulate protein expression of p21, rescue experiments was performed. cck8 assay displayed that AGS cells with elevated circRHOBTB3 plus miR-654-3p grown slower than miR-654-3p group (Fig. 5a, b), and consistent results was reproduced in HGC27 cells. Likewise, the colony assay showed that overexpressed circRHOBTB3 GC cells together with miR-654-3p exerted less cloned cells than GC cells transfected with miR-654-3p (Fig. 5c). Moreover, cell cycle distribution was analyzed by flow cytometry in GC cells and results suggested that miR-654-3p combined with circRHOBTB3 reversed miR-654-3p-induced cell cycle progression, resulting in more cells ceased in G1 phase (Fig. 5d, e). Then, to see whether circRHOBTB3 could restore the expression levels of p21 inhibited by miR-654-3p, p21 was examined using western blot, and the brightness of the protein band was observed to be stronger in circRHOBTB3 + miR-654-3p group than in miR-654-3p group (Fig. 5f). These results indicated that circRHOBTB3 reversed miR-654-3p-induced enhancement of GC cell growth, and could restore the expression of miR-654-3p target p21 at protein levels, forming the circRHOBTB3/miR-654-3p/p21 regulating axis (Fig. 5h).

### Stable overexpression of circRHOBTB3 inhibits GC growth in vivo by targeting p21

**Fig. 6:**
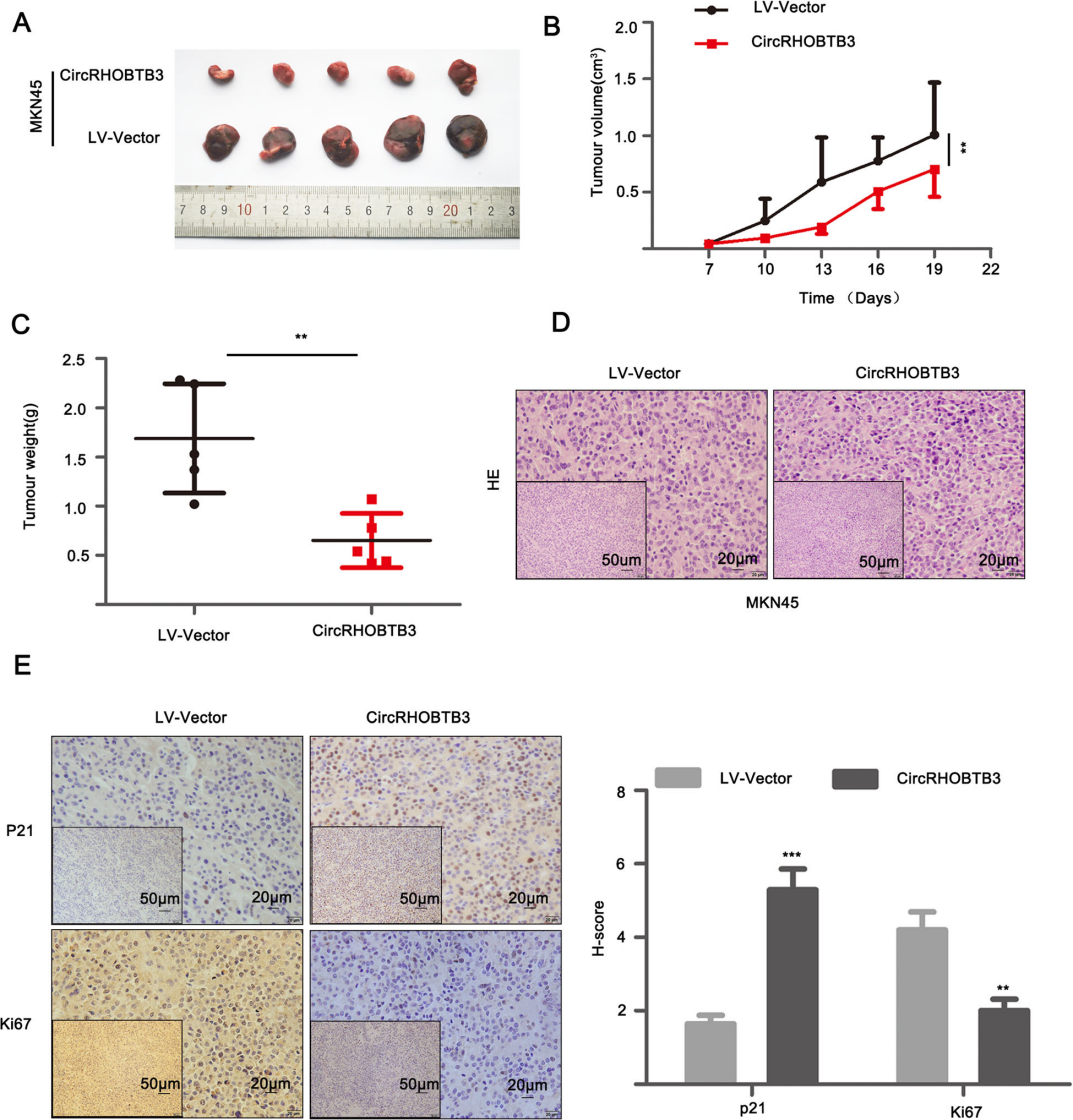
CircRHOBTB3 inhibited tumor growth in vivo. **a** Representation image of xenograft tumor in nude mice (*n* = 5). **b** Overexpressed circRHOBTB3 suppressed tumor growth. **c** Analysis of tumor weight after xenograft tumor removed. **d** H&E-stained paraffin-embedded tumor obtained from xenograft tumor. **e** IHC staining for p21 and ki67 in xenograft tumor and p21/ki67 expression was evaluated by expression score. Representative bottom pictures were local magnification of top pictures. *p < 0.05, **p < 0.01, ***p < 0.001

To assess whether overexpression of circRHOBTB3 affects tumor growth in vivo, MKN45 transfected with elevated circRHOBTB3 lentivirus or control vector was injected subcutaneously into nude mice. Haematoxylin and eosin (H&E) staining was applied to confirm the histopathological features of tumors harvested. Tumor volume shrink and reduced average tumor weight were observed in overexpressed circRHOBTB3 group compared with control group (Fig. 6a-d). Immunohistochemistry (IHC) demonstrated that the expression of ki67 proliferation antigen was significantly weakened in overexpressed circRHOBTB3 xenografts. Moreover, the staining of p21 was observed stronger than control group (Fig. 6e). Therefore, circRHOBTB3 could present tumor-suppressing property to inhibit cell growth and produce cell cycle arrest.

## Discussion

CircRNAs has been long considered to be molecular flukes or by-products of splicing ever since they were observed in eukaryotic cells by electron microscopy almost 40 years ago [10, 35]. However, the understanding of circRNAs has gradually changed with the application and development of the next-generation sequencing. A large number of circRNAs were detected in various animal samples and multiple cell lines, and many of them were found to be highly stable and abundantly expressed, based on which, subsequent studies demonstrated that circRNAs were dysregulated in diverse cancers including colorectal cancer [13, 30], hepatocellular carcinoma [18], basal cell carcinoma [36], laryngeal cancer [37], bladder cancer [20]. It has been reported that circRNAs could serve as the prognostic biomarkers for many cancers. For example, elevation of circHIPK3 was correlated with poor prognosis in bladder cancer and colorectal cancer [20, 30]. Patients with lower circ-ITCH expression had a poorer prognosis in bladder cancer [22], and higher levels of circPRCKI displayed as a worse progression marker in lung adenocarcinoma [23]. Whereas, their roles in GC remained largely unknown. In this study, we found that circRHOBTB3 was frequently low-expressed in GC tissues compared with adjacent normal tissues. The low expression of circRHOBTB3 was correlated with tumor stage and provided the poor prognosis of GC patients. Astoundingly, clinical correlation analyses showed that tumor size was not associated with circRHOBTB3 expression, which was not consistent with its functional effects in GC. One possible reason might be that the number of GC specimens was too few. Thus, it’s necessary to analyze the relationship between circRHOBTB3 and clinicopathological parameter using more GC tissue samples in the future study.

CircRNAs could act as oncogenic or tumor suppressive factors in GC. CircFAT1 displayed inhibiting effects in GC [38], repressing proliferation and invasion of GC cells, but circPVT1/circDLST enhanced the malignancy of GC [19, 39]. Currently, we identified a functional role of circRHOBTB3 in GC cells and validated that re-expression of circRHOBTB3 suppressed the growth of GC in vitro and vivo and arrested cell cycle in G_1_ phase, while knockdown of circRHOBTB3 produced contrary effects. These results indicated that circRHOBTB3 might serve as a potential tumor suppressive marker in GC.

Recently, increasing evidences have shown that circRNAs functions as sponge for miRNAs to affect tumorous biological process [40]. For instance, circSMARCA5 could act as sponge for miR-17-3p and miR-181b-5p to inhibit the growth and metastasis in hepatocellular carcinoma [41]. CircPVT1 interacted with miR-125b to exert proliferative effect in GC [19]. Herein, we found that circRHOBTB3 contained the binding sites of miR-654-3p predicted by bioinformatic website circinteractome and verified there was direct binding relationship between circRHOBTB3 and miR-654-3p using biotinylated-nucleic probe pull down assay and luciferase reporter experiments, which suggested that circRHOBTB3 might serve as a sponge for miR-654-3p to inhibit the proliferation of GC.

MiRNAs have been shown to be a large family of gene regulator that negatively regulate their target mRNAs in a sequence-specific manner and its role in cancers have been extensively studied [31, 42, 43]. Numerous evidences have confirmed that miRNAs play essential roles in multiple biological process related to cancer, including cell differentiation, proliferation, tumorigenesis, angiogenesis, invasion, and metastasis [44, 45]. As described, miR-654-3p was confirmed to target p21 mRNA and induced its protein downregulation in HEK293 cells [46]. Previous studies have demonstrated that p21 could suppress multiple tumorous proliferation by targeting cyclin-dependent kinase (CDK) complexes [46, 47]. However, whether miR-654-3p could regulate the expression of p21 and biological function in GC cells remained unknown. In the present study, we proved that miR-654-3p was up-regulated in GC tissues and cells, and promoted the proliferation of GC cells. Additionally, we also confirmed that miR-654-3p could inhibit the protein expression of p21 in GC cells using western blot, whereas, circRHOBTB3 enhanced the protein expression of p21. Further reversed experiments were conducted and we found that circRHOBTB3 inhibited the activity of miR-654-3p, and thus, reversed the miR-654-3p induced proliferation of GC cells and up-regulated the protein expression of p21 indirectly. Hence, we validated that circRHOBTB3 could inhibit the proliferation of GC by sponging miR-654-3p to up-regulate the protein expression of p21. One thing we have to pay attention is that not all circRNAs can function as “miRNA sponges” [48]. Intronic circRNAs and exon-intronic circRNAs, which predominantly localize in nucleus with lacking enrichment for miRNA target sites, have been reported to modulate parental genes expression via RNA-RNA interaction [29, 49]. Meanwhile, some circRNAs, such as circMbl, circFmn, circDMD, can strongly bind to cognate linear transcripts to prevent mRNA from translation and finally lead to the reduction in protein levels [48, 50]. This process is also termed as “mRNA trap”. Thus, diverse functions of circRNAs in GC need to be further explored.

## Conclusions

Our study confirmed that circRHOBTB3 was frequently down-regulated in GC and low expression of circRHOBTB3 was correlated with tumor stage and patients’ unfavorable prognosis. Mechanistically, circRHOBTB3 could serve as a sponge molecule for miR-654-3p, and then rescued the expression of miR-654-3p-inbiting protein p21, finally suppressed GC cell growth. Our findings might provide new insight into GC development and provided a novel potential strategy for GC treatment.

## Supplementary information

**Additional file 1: Figure S1.** RNA FISH was carried out to detect circRHOBTB3’s subcellular localization in AGS *cells.* **Figure S2.** Silencing of circRHOBTB3 promoted proliferation and progression of cell cycle in HGC27 cells. **Figure S3.** CircRHOBTB3 served as sponge of miR-654-3p. **Figure S4.** CircRHOBTB3 modulated the expression of endogenous miR-654-3p target p21.

**Additional file 2: Table S1.** Primers and RNA sequences used in this study.

## Data Availability

The datasets used and analysed during the current study are available from the corresponding author on reasonable request.

## Abbreviations

CDK: Cyclin-dependent kinase
ceRNA: Competing endogenous RNA
circRHOBTB3: Circular RNA RHOBTB3
circRNAs: Circular RNAs
FISH: Fluorescence in situ hybridization
GC: Gastric cancer
gDNA: Genomic DNA
H&E: Hematoxylin and eosin
IHC: Immunohistochemistry
miRNA: MircroRNA
qRT-PCR: Quantitative real-time polymerase chain reaction
