# Supplementary figures and images for: Circular RNA circRHOBTB3 acts as a sponge for miR-654-3p inhibiting gastric cancer growth
Source: J Exp Clin Cancer Res. 2020 Jan 13;39:1. doi: 10.1186/s13046-019-1487-2 (PMC6956561; doi:10.1186/s13046-019-1487-2)

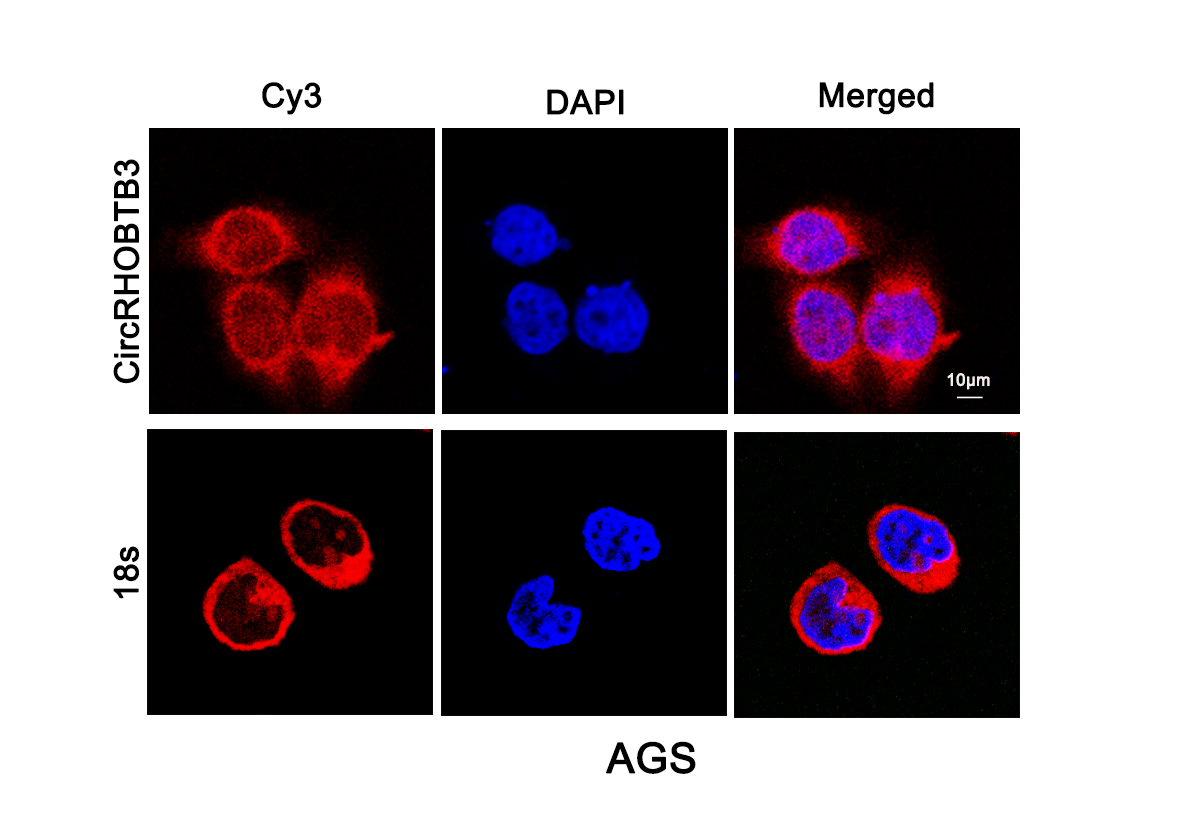

Supplement: Supplementary file 1 — Additional file 1: Figure S1. RNA FISH was carried out to detect circRHOBTB3’s subcellular localization in AGS cells. Figure S2. Silencing of circRHOBTB3 promoted proliferation and progression of cell cycle in HGC27 cells. Figure S3. CircRHOBTB3 served as sponge of miR-654-3p. Figure S4. CircRHOBTB3 modulated the expression of endogenous miR-654-3p target p21. [file 13046_2019_1487_MOESM1_ESM.zip › S1.tif]

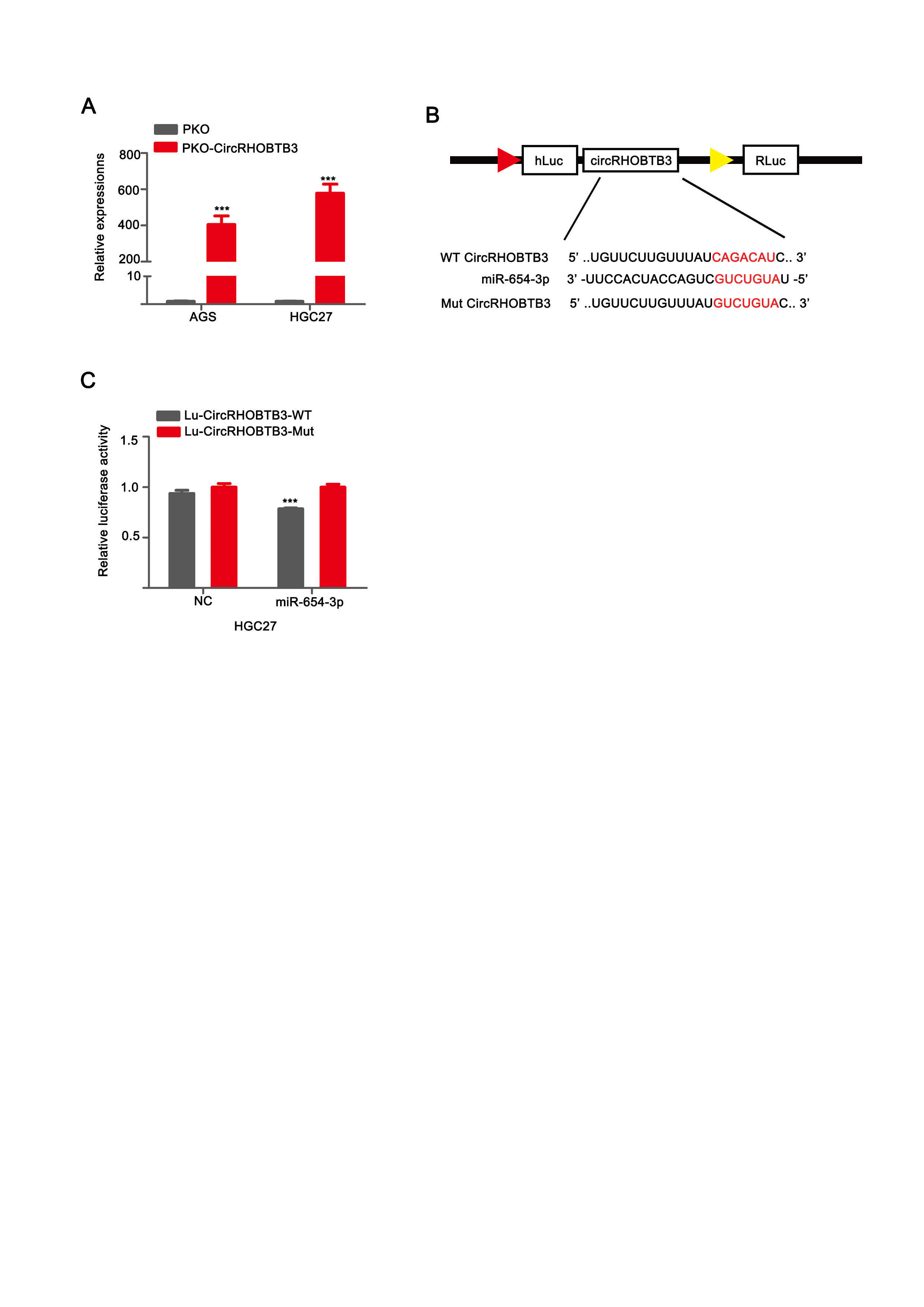

Supplement: Supplementary file 1 — Additional file 1: Figure S1. RNA FISH was carried out to detect circRHOBTB3’s subcellular localization in AGS cells. Figure S2. Silencing of circRHOBTB3 promoted proliferation and progression of cell cycle in HGC27 cells. Figure S3. CircRHOBTB3 served as sponge of miR-654-3p. Figure S4. CircRHOBTB3 modulated the expression of endogenous miR-654-3p target p21. [file 13046_2019_1487_MOESM1_ESM.zip › S2.tif]

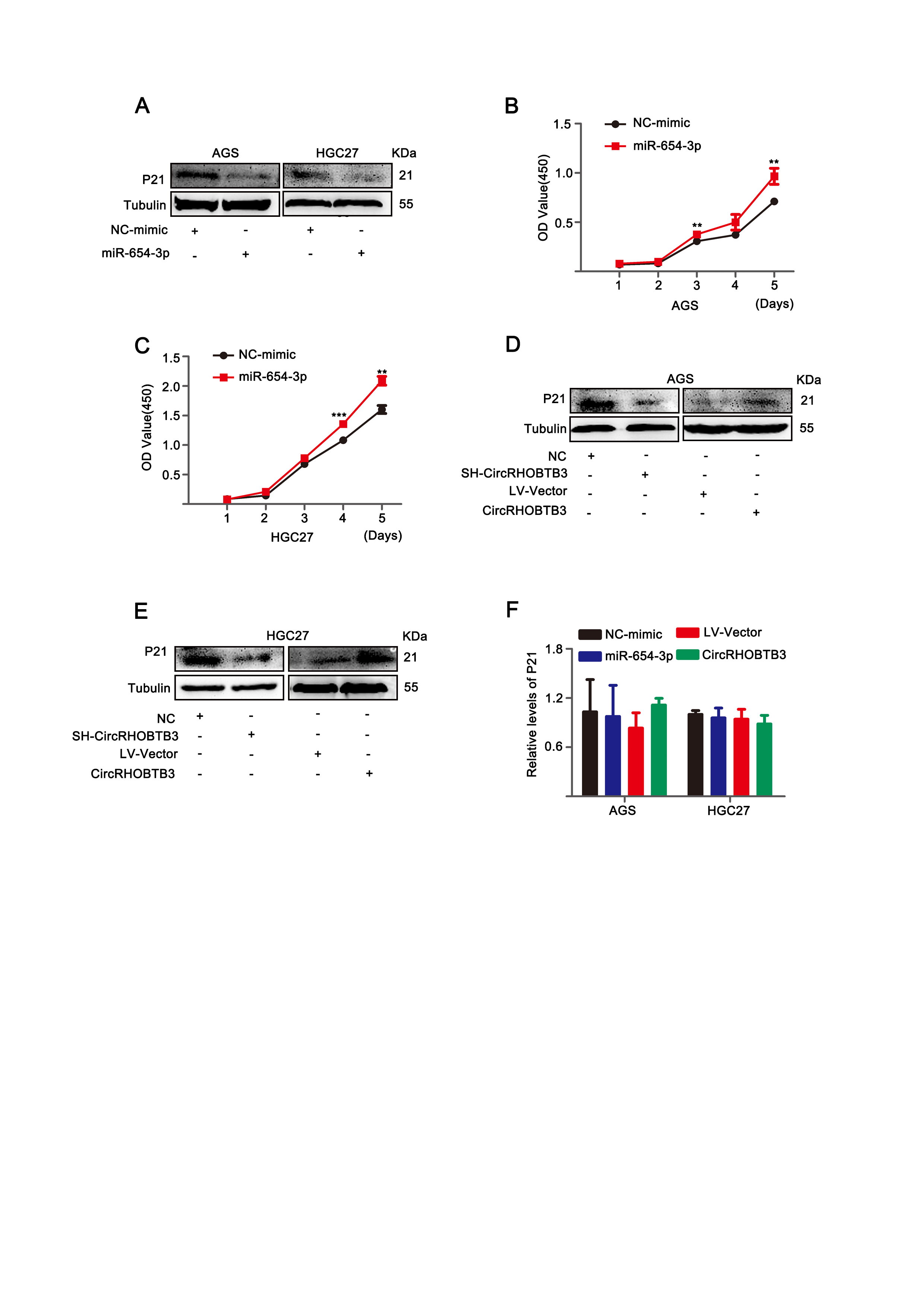

Supplement: Supplementary file 1 — Additional file 1: Figure S1. RNA FISH was carried out to detect circRHOBTB3’s subcellular localization in AGS cells. Figure S2. Silencing of circRHOBTB3 promoted proliferation and progression of cell cycle in HGC27 cells. Figure S3. CircRHOBTB3 served as sponge of miR-654-3p. Figure S4. CircRHOBTB3 modulated the expression of endogenous miR-654-3p target p21. [file 13046_2019_1487_MOESM1_ESM.zip › S3.tif]

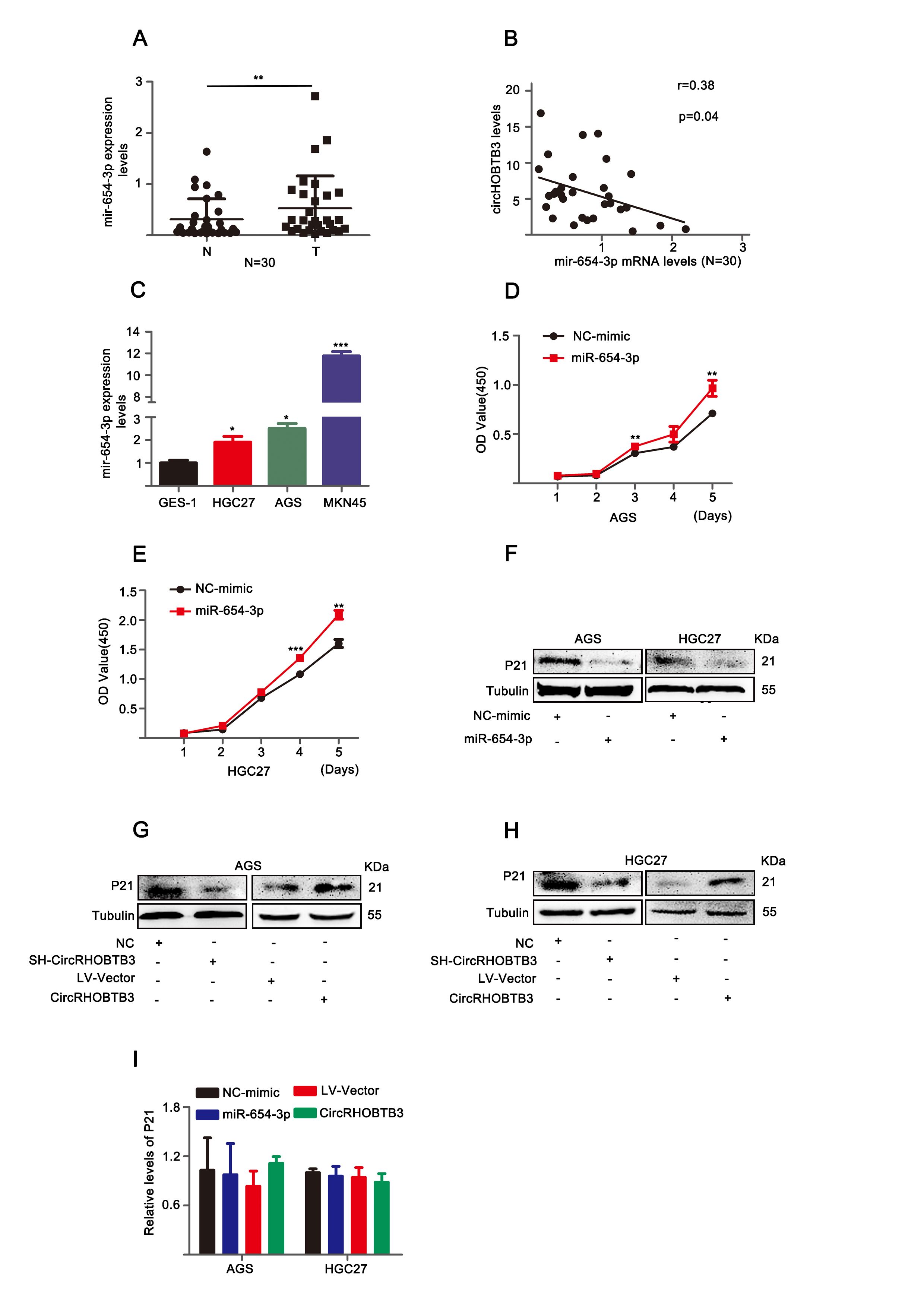

Supplement: Supplementary file 1 — Additional file 1: Figure S1. RNA FISH was carried out to detect circRHOBTB3’s subcellular localization in AGS cells. Figure S2. Silencing of circRHOBTB3 promoted proliferation and progression of cell cycle in HGC27 cells. Figure S3. CircRHOBTB3 served as sponge of miR-654-3p. Figure S4. CircRHOBTB3 modulated the expression of endogenous miR-654-3p target p21. [file 13046_2019_1487_MOESM1_ESM.zip › S4.tif]
