# Supplementary material for: Circular RNA circRHOBTB3 acts as a sponge for miR-654-3p inhibiting gastric cancer growth
Source: J Exp Clin Cancer Res. 2020 Jan 13;39:1. doi: 10.1186/s13046-019-1487-2 (PMC6956561; doi:10.1186/s13046-019-1487-2)
Supplement: Supplementary file 2 — Additional file 2: Table S1. Primers and RNA sequences used in this study. [file 13046_2019_1487_MOESM2_ESM.doc]

**Circular RNA circRHOBTB3 acts as a sponge for miR-654-3p inhibiting gastric cancer growth**

Guangxu Deng^1*^, Tingyu Mou^1*^, Jiayong He^1*^, Da Chen^1*^, Daojun Lv^4^, Hao Liu^1^, Jiang Yu^1^, Shuang Wang^2,3#^, Guoxin Li^1#^

**Supplementary data**

**Figure S1*.*** RNA FISH was carried out to detect circRHOBTB3’s subcellular localization in AGS *cells.*

**Figure S2*. Silencing of circRHOBTB3 promoted proliferation and progression of cell cycle in HGC27 cells.*** (**A**) The interfering efficacy of circRHOBTB3 or linear RHOBTB3 mRNA were quantified by qRT-PCR in AGS and HGC27 cells transfected with specifically targeting circRHOBTB3 small interfering RNA (siRNA) and its negative control (**B**) CircRHOBTB3 levels and RHOBTB3 mRNA levels were detected after transfected with lentivirus-vector or lentivirus-SH-circRHOBTB3 in AGS and HGC27 cell lines. (**C**) The relative levels of circRHOBTB3 and his linear RHOBTB3 mRNA were detected by qRT-PCR after transfected with LV-Vector or LV-circRHOBTB3. (**D**) cck8 assay was conducted in stable silenced circRHOBTB3 HGC27 and control group. (**E**) The colony formation assay was performed to evaluate colony formation ability of silencing circRHOBTB3 in HGC27 cells. (**F**) Edu assay was performed in HGC27 cells after knockdown of circRHOBTB3. (**G**) Cell cycle analysis in HGC27 cells with silencing circRHOBTB3 using flow cytometry. The data was expressed as the mean ± SD and reproduced in three independent experiments. **p*<0.05, ***p*<0.01, ****p*<0.001.

**Figure S3*. CircRHOBTB3 served as sponge of miR-654-3p****.* (**A**) The expressed efficacy of circRHOBTB3 was tested using qRT-PCR in HGC27 and AGS cell lines after transfected with plasmid-Vector or plasmid-circRHOBTB3. (**B**) Schematic of circRHOBTB3 wild-type (WT) and mutant (Mut) luciferase reporter vectors. (**C**) Luciferase activity was detected in HGC27 cells co-transfected with luciferase reporter containing circRHOBTB3 sequences with wild type and mutant binding site of miR-654-3p and the mimic of miR-654-3p or control. (**D**) Fluorescence in situ hybridization was performed to examine the co-location between circRHOBTB3 and miR-654-3p in HGC27 cell lines. The data was expressed as the mean ± SD and reproduced in three independent experiments. **p*<0.05, ***p*<0.01, ****p*<0.001.

**Figure S4*. CircRHOBTB3 modulated the expression of endogenous miR-654-3p target p21.*** (**A**) The relative expressions of miR-654-3p were quantified by qRT-PCR in 30 pairs of GC tissues and noncancerous samples. (**B**) The relationship between circRHOBTB3 and miR-654-3p was evaluated by Pearson’s correlation coefficients in GC and normal tissues(n=30). (**C**) The levels of miR-654-3p was testing by qRT-PCR in GC cell lines. (**D, E**) MiR-654-3p affected the proliferation of AGS and HGC27 cells using cck8 assay. (**F**) Western blot was performed to examine the expression of p21 in AGS and HGC27 cells transfected with miR-654-3p and NC-mimic. (**G, H**) CircRHOBTB3 regulated the protein expression of p21 in AGS and HGC27 cells by western blot. (**I**) qRT-PCR detection for p21 mRNA expression in AGS and HGC27 cells transfected with miR-654-3p or NC-mimic or LV-Vector or LV-circRHOBTB3. The data was expressed as the mean ± SD and reproduced in three independent experiments. **p*<0.05, ***p*<0.01, ****p*<0.001.

**Table S1 primers and RNA sequences used in this study**

| **List of oligonucleotide**  **sequences** | **5’→3’** |
| --- | --- |
| **Primers for Real-time PCR and RT-PCR** | |
| circRHOBTB3—qF | GAAGTTGAAAGATTCTGGGGA |
| circRHOBTB3—qR | ACTGGCAGCAGAACAGCAAG |
| RHOBTB3—qF | ATAAGCCGATGCTTGCCGAT |
| RHOBTB3—qR | GCTGCCATCACTTCACAACG |
| circRHOBTB3—RT—F | TCCAGTATCATCCGAACTACCC |
| circRHOBTB3—RT—R | CTTACAGCGCAGAGAACGATC |
| RHOBTB3—RT—F | ATAAGCCGATGCTTGCCGAT |
| RHOBTB3—RT—R | GCTGCCATCACTTCACAACG |
| GAPDH—F | GGAGCGAGATCCCTCCAAAAT |
| GAPDH—R | GGCTGTTGTCATACTTCTCATGG |
| 18SrRNA—F | ACACGGACAGGATTGACAGA |
| 18SrRNA—R | GGACATCTAAGGGCATCACA |
| circN4BP2L2—qF | CGAAAGAAGATTGCTCAGATGTT |
| circN4BP2L2—qR | GCCATCACGATTCTGACCAA |
| N4BP2L2—qF | TCGTGCTCTTTGGGAGTCAC |
| N4BP2L2—qR | CCATTGGAACGCCTGGAGAT |
| circRELL1—qF | TAGTGGGGCTGAAACCGTC |
| circRELL1—qR | GCTGTTATCTGCTACCATCGC |
| RELL1—qF | TCCAGAACAGAGACGACCCC |
| RELL1—qR | ACGCCAAAGAGACCCATGAT |
| circFAM120A—qF | AAGCCTATGTCATTTCATCCACC |
| circFAM120A—qR | GGCACTGAAATAGTAGGGGATG |
| FAM120A—qF | TTCTGTCGGAGGACCAGCTA |
| FAM120A—qR | CAGCATAAGGAGGTCTGCCC |
| circFNDC3B—qF | TAAGAAAACAGAGCGACGAGC |
| circFNDC3B—qR | GGTGAGTCAGATAGGGAGGGA |
| FNDC3B—qF | TCCAGAAACCACCTACCGGA |
| FNDC3B—qR | TCTAGCCTAGGAGGCAAGGG |
| p21—qF | CACTGGAGGGTGACTTCGC |
| p21—qR | GCCTCCTCCCAACTCATCC |
| U6—qF | CTCGCTTCGGCAGCACA |
| U6—qR | AACGCTTCACGAATTTGCGT |
| hsa-miR-1200-5P—qF | CTCCTGAGCCATTCTGAGCCT |
| hsa-miR-600-3P—qF | ACTTACAGACAAGAGCCTTGCTC |
| hsa-miR-1265-5P—qF | CAGGATGTGGTCAAGTGTT |
| hsa-miR-619-3P—qF | GACCTGGACATGTTTGTGCC |
| hsa-miR-654-3p—qF | TATGTCTGCTGACCATCACCTT |
| hsa-miR-1305—qF | TTTTCAACTCTAATGGGAGAG |
| hsa-miR-548p—qF | TAGCAAAAACTGCAGTTACTTT |
| hsa-miR-494—qF | TGAAACATACACGGGAAAC |
| hsa-miR-570—qF | CGAAAACAGCAATTACCTTT |
| hsa-miR-586—qF | TATGCATTGTATTTTTAGGTCC |
| hsa-miR-224 | CTGGTAGGTAAGTCACTA |
| **siRNAs** | |
| si-circRHOBTB3-1 | CACCAGGAAAGAAAAAAUGTT |
| si-circRHOBTB3-2 | GGAAAGAAAAAAUGCCUGUTT |
| NC-siRNA | UUCUCCGAACGUGUCACGUTT |
